# Association between the rs615563 variant of PCSK9 gene and circulating lipids and Type 2 diabetes

**DOI:** 10.1186/s13104-021-05723-4

**Published:** 2021-08-11

**Authors:** Samira Taghizadeh Jazdani, Hajieh Bibi Shahbazian, Bahman Cheraghian, Mohammad Taha Jalali, Narges Mohammadtaghvaei

**Affiliations:** 1 Health Research Institute, Diabetes Research Center, Ahvaz Jundishapur University of Medical Sciences, Ahvaz, Iran; 2 Department of Laboratory Sciences, Faculty of Paramedicine, Ahvaz Jundishapur University of Medical Sciences, Ahvaz, Iran; 3 Department of Epidemiology and Biostatistics, School of Health, Ahvaz Jundishapur University of Medical Sciences, Ahvaz, Iran; 4 Hyperlipidemia Research Center, Ahvaz Jundishapur University of Medical Sciences, Ahvaz, Iran

**Keywords:** Type 2 diabetes, LDL cholesterol, rs615563, PCSK9, PCR–RFLP

## Abstract

**Objective:**

Many different genetic variants of proprotein convertase subtilisin kexin 9 (PCSK9) are related to the serum levels of cholesterol and LDL cholesterol (LDL-C). The rs615563 variant of PCSK9 (a gain-of-function mutation) is associated with increased triglycerides and cholesterol levels, but its association with the incidence of diabetes is not well defined. This study aimed to investigate the relationship between the PCSK9 rs615563 variant with the incidence of type 2 diabetes. The data reported in this study are based on subsamples from a 5-year (2009–2014) cohort study of the adult population (590 subjects) aged 20 years and older. The rs615563 polymorphism was genotyped using polymerase chain reaction (PCR) followed by restriction fragment length polymorphism (RFLP) analysis.

**Results:**

The distribution of PCSK9 rs615563 genotypes was not significantly different between the diabetic and non-diabetic individuals. The incidence of diabetes after five-years of follow-up was not different between the genotypes. Our findings also showed no significant relationship between this polymorphism and serum lipid parameters. The data extracted from our cohort study do not support the findings that the gain-of-function mutations of PCSK9 predispose to the incidence of type 2 diabetes.

**Supplementary Information:**

The online version contains supplementary material available at 10.1186/s13104-021-05723-4.

## Introduction

The ninth member of the proprotein convertase subtilisin kexin (PCSK9) family was identified in early 2003. PCSK9 gene, also known as neural apoptosis-regulated convertase 1 (NARC1), is located on chromosome 1p32.3. This gene encodes an autocatalytic enzyme predominantly expressed in hepatocytes and is released directly into the circulation. Extrahepatic tissues such as the intestine, kidney, brain, blood vessels, and pancreas express PCSK9 to a lesser extent [1, 30].

PCSK9 has a critical role in the regulation of low-density lipoprotein cholesterol levels. PCSK9 binds to the hepatic LDL receptors and promotes their degradation, leading to decreased number of LDL receptors on hepatocytes [12]. Several different genetic variants of PCSK9 have been reported in various populations, including gain-of-function (GOF) and loss-of-function (LOF) variants [6, 8, 11, 19, 20]. Gain-of-function PCSK9 variants can increase plasma LDL cholesterol levels by accelerating LDL receptors degradation, and in contrast, loss-of-function variants of PCSK9 can decrease plasma LDL cholesterol levels by reducing LDL receptors degradation [16]. PCSK9 is known as a therapeutic target to control LDL cholesterol levels due to the significant role of PCSK9 on plasma LDL cholesterol clearance [34]. The relationship between plasma PCSK9 concentration and diabetes mellitus has been of note in some previous studies. The majority of the results showed that diabetes was significantly associated with increased plasma levels of PCSK9 [7, 21, 23], but there are also contradictory results showing an inverse relationship [5, 9]. This controversy may be explained by the fact that PCSK9 is also expressed in the pancreas, and this locally produced PCSK9 may play the main role in insulin secretion impairment. In the PCSK9 deficiency condition, although the plasma cholesterol level is decreased, cholesterol accumulation in the β-cells increases, leading to β-cell dysfunction and diabetes [13].

Genetic studies have shown that PCSK9 alleles associated with reduced LDL cholesterol levels increase the risk of developing type 2 diabetes mellitus (T2DM) [28]. In contrast, the alleles associated with the increased LDL cholesterol may reduce the risk of T2DM. Regarding the fact that PCSK9 is the main determinant factor in regulating plasma LDL cholesterol level and the genetic variants of PCSK9 may predispose to the incidence of T2DM, this study investigated the relationship between the PCSK9 rs615563 genotypes (a GOF allele) with the levels of circulating lipids and the incidence of diabetes on subsamples from a 5-year (2009–2014) cohort study. The current study results are significant because worldwide regulatory agencies have cleared PCSK9 inhibitors as cholesterol-lowering drugs.

## Main text

### Methods

#### Study population

The data reported in this study are based on subsamples from a 5-year (2009–2014) cohort study of the adult population of Ahvaz, Iran (590 subjects). The detailed information about the study population is described in Additional file 1.

#### Biochemical analyses

Fasting blood samples were drawn from the antecubital vein and then centrifuged at 2500 rpm for 10 min to separate serum. The detailed procedures are described in Additional file 1.

#### Genotyping of rs615563 variants

The rs615563 was genotyped using polymerase chain reaction (PCR) -restriction fragment length polymorphism (RFLP). The amplified product of PCR was a 365 bp fragment which was digested to three fragments of 32, 333, and 365 bp. The PCR products with homozygous allele (GG) were digested into two bands of 333 and 32 bp, while the heterozygous (GA) yielded three bands of 365, 333, and 32 bp, and finally, the homozygous allele (AA) presented one band of 365 bp. The detailed procedure is described in Additional file 1.

#### Statistical analysis

Statistical analysis was performed with the SPSS, version 22. Quantitative data were reported as mean ± SD or median (interquartile range), and qualitative data were reported as frequency and percentage.

One-way ANOVA and Kruskal–Wallis tests were conducted for quantitative variables with normal distribution or non-normal distribution, respectively, to define the relationship between the variables with more than two groups. Cluster analysis (ANCOVA) was used to control the confounding factors in the groups. We performed logistic regression analysis with and without adjustment for age and sex and body mass index (BMI). Odds ratios (OR) were calculated for different genotypes with different outcomes. A non-conditional logistic regression model for odds ratio with confidence intervals was used to investigate the relationship between the genotypes and the disease to control the possible confounding factors. A p-value < 0.05 was considered statistically significant.

### Results

We assessed the association of PCSK9 rs615563 genotypes with the levels of FBS, BMI, lipid parameters, including total cholesterol, HDL cholesterol, LDL cholesterol, non-HDL cholesterol, triglyceride, and liver enzyme levels (AST and ALAT). The results of genetic analysis and the frequency of the rs615563 variants in 590 subjects showed that 328 subjects had homozygous GG genotype, 205 subjects had heterozygote genotype GT, and 57 subjects showed homozygous TT genotype. When we adjusted our results for age, sex, and BMI, we did not find any significant association between the PCSK9 rs615563 variants and our variables in non-diabetic and diabetic participants (Tables 1, 2).

**Table 1:** Associations between the PCSK9 rs615563 variant and, FBS, lipid homeostasis variables and liver enzyme levels in 500 non-diabetic participants

Clinical data	N	Mean/median data level by rs615563 genotype			p value	Adjusted p-value^a^
		GG	GA	AA		
Sex (M/F)	197/303	105/165 38.9 / 61.1%	67/116 36.6 / 63.4%	25/22 53.2 / 46.8%	-	-
Age (years)	499	41.52 ± 14.97	39.34 ± 14.96	40.09 ± 12.37	0.296	-
BMI (kg/m2)	495	26.39 ± 14.7	27.18 15.62 ±	27.10 ± 18.83	0.380	-
FBS (mg/dl) (IQR)	500	92 (85–100)	92 (85–98)	92 (82–99)	0.649	0.72
TC (mg/dL)	500	184.07 ± 38.04	181.53 ± 38.15	185.80 ± 36.97	0.701	0.8
HDL-C (mg/dL)	500	46.72 ± 10.23	46.77 ± 9.09	45.60 ± 9.47	0.745	0.96
Non-HDL (mg/dL)	500	136.06 ± 35.24	133.18 ± 35.59	139.53 ± 37.55	0.488	0.66
LDL-C (mg/dL)	500	107.70 ± 33.45	105.40 ± 36.80	108.27 ± 13	0.760	0.81
TG (mg/dl) (IQR)	500	115 (78.5–163)	103 (72–146)	119 (72–195)	0.137	0.36
ALT (U/I) (IQR)	428	12 (9–17.75)	13 (9–18)	12 (8–15)	0.465	0.7
AST (U/I) (IQR)	428	27.5 (23–35)		28 (23–34)	0.894	0.64

Data are expressed as mean ± SD or median (IQR)

*IQR* interquartile range, *FBS*, Fasting blood sugar, *ALAT* alanine aminotransferase, *AST *aspartate aminotransferase, *HDL-C* HDL cholesterol, *LDL-C* LDL-cholesterol, *TC* total cholesterol, *TG* triglyceride

^a^Adjusted for sex, age, BMI

**Table 2:** Associations between the PCSK9 rs615563 variant and, FBS, lipid homeostasis variables and liver enzyme levels in patients with diabetes

Variables	Genotype			p-value^a^
	GG (n = 58)	GA (n = 22)	AA (n = 10)	
Sex (M/F)	24/34	13/9	5/5	0.35
Age (years)	56.5 ± 13	54.36 ± 10.5	53.5 ± 13	0.57
BMI (kg/m^2^)	28 ± 5	26.82 ± 4.5	30.5 ± 7	0.32
FBS (mg/dL)*	176 (136.-213.)	169.5 (134–193)	158 (128–177.5)	0.2
TC (mg/dL)*	199.87 ± 41.41	194.6 ± 45.54	198.95 ± 38.95	0.95
TG (mg/dL)*	155 (116–214)	140 (92–246)	162 (124.25–193)	0.46
HDL-C (mg/dL)*	45.8 ± 8.84	44.54 ± 11	46.3 ± 9.8	0.75
Non-HDL (mg/dL)*	153.36 ± 38.93	152.72 ± 37.92	148.3 ± 43.38	0.7
LDL-C (mg/dL)*	117.65 ± 39.40	109.59 ± 41.85	118.70 ± 52.03	0.9
ALAT (U/I)*	15 (10–20)	15 (10–22.5)	10 (8–19)	0.29
AST (U/I)*	29(23–36)	28.5(24–36.25)	30 (22.5–33.5)	0.82

^a^Adjust for sex, age, BMI

In the case–control analysis, we did not find any significant association between the PCSK9 rs615563 and type 2 diabetes [GG OR (95% CI) 1.78 (0.79, 4.44); p = 0.16], [AA OR (95% CI) 1.48 (0.84, 2.61); p = 0.17] and the incidence of type 2 diabetes over the 5 years of follow-up [GG OR (95% CI) 1.55 (0.85, 4.39); p = 0.16], [AA OR (95% CI) 1.72 (0.84, 2.61); p = 0.26] (Table 3).

**Table 3:** Type 2 diabetes case–control and incidence analysis rs615563 (G/A)

	Non-Diabetic	Diabetic	OR (%95 CI)^a^	p-value
Type 2 diabetes case–control analysis (n = 590)				
GG	270 (82.3%)	58 (17.7%)	1.78 (0.79–4.44)	0.16
GA	183 (89.3%)	22 (10.7%)	Reference	
AA	47 (82.5%)	10 (17.5%)	1.48(0.84–2.61)	0.17
Allele frequency				
G	72.3%	77%		
A	27.7%	23%		
Type 2 diabetes incidence analysis (n = 580)				
GG	270 (83.6%)	53 (16.4%)	1.55 (0.85–4.39)	0.16
GA	183 (90.6%)	19 (9.4%)	Reference	
AA	47 (85.5%)	8 (14.5%)	1.72 (0.84–2.61)	0.26
Allele frequency				
G	72.3%	78%		
A	27.7%	22%		

^a^OR from a logistic regression model adjusted for age, sex and BMI

### Discussion

To our knowledge, this is the first study investigating the association of the PCSK9 rs615563 polymorphism and the incidence of diabetes. According to the results, there was no significant difference in the distribution of rs615563 alleles among non-diabetic and type 2 diabetic patients in the case–control group. There were no significant differences in FBS levels of homozygous and heterozygous subjects (Table 1).

In the case–control study, we showed that the incidence of type 2 diabetes in rs615563 variants did not increase during a five-year follow-up.

PCSK9 by degrading LDL receptor on the cells surface represents a crucial regulator of LDL receptor [2, 22, 33]. Targeting PCSK9 with monoclonal antibodies demonstrates the newest and most promising pharmacological tool for the treatment of hypercholesterolemia. Different common and rare gene variants influence PCSK9 function. Its gain-of-function mutations have been associated with hypercholesterolemia, and loss-of-function mutations result in low LDL cholesterol levels [1, 10, 31]. PCSK9 rs615563, a gain of function mutation, is associated with elevated LDL cholesterol levels. In a study conducted by Tao Guo et al., positive correlations were observed between total plasma cholesterol and triglyceride, with PCSK9 rs615563 in patients with hyperlipidemia [18]. In another study, Guo et al. demonstrated an association of the rs615563 with increased triglyceride in two healthy Chinese populations [17]. In our study, we did not find any associations between total cholesterol and triglyceride with PCSK9 rs615563. The differences in the genetic background may partly explain the reason for these discrepancies. Guo et al. included healthy individuals from two different populations, Han and Jing, from China in their study. While the Han population is the largest nationality in china, Jing is the smallest one and is a relatively conservative and isolated population with low genetic heterogeneity [17]. Therefore, genetic background and mutations in other lipid-related genes in this population were most probably different from our studied population. Furthermore, the phenotypic expression of the PCSK9 mutations is affected by several environmental factors, including dietary habits, physical activity, and lifestyles that are different between the populations.

Our results are consistent with a recent study by Zamarrón-Licona et al. in healthy individuals and subclinical atherosclerosis patients. No significant association between total plasma cholesterol and triglyceride, with PCSK9 rs615563, was found in this study, and interestingly, the rs615563 polymorphism in the control group was associated with a decreased risk of hypertriglyceridemia [32].

Genetic studies of the effects of gene variants in a wide range have shown a link between the gene variants with decreased LDL cholesterol and increased risk of type 2 diabetes [15, 25]. According to these findings, patients with autosomal familial hypercholesterolemia caused by a mutation in the LDL receptor and apolipoprotein B receptor are diagnosed with type 2 diabetes 50% less than those without these mutations [3]. There are ample LDL receptors on the cell surface of pancreatic beta-cells that play a pivotal role in the uptake and homeostasis of islet cholesterol. Experimental evidence using PCSK9-deficient mice revealed that elevated expression of LDL receptor is associated with a decrease in LDL cholesterol level in the circulation, impaired glucose tolerance, and pancreatic islet abnormalities [13]. In the PCSK9 deficiency state, excessive cholesterol accumulation affects pancreatic beta-cell function that may reduce its ability to secrete insulin in response to glucose [13]. These findings were further advocated in subjects with a PCSK9 loss of function. In line with this evidence, Mendelian randomization studies also confirmed the association of PCSK9 variation with the increased risk of type 2 diabetes [29].

In contrast to these findings, we did not find an association between PCSK9 rs615563 genotypes and the diabetic status or its incidence in the current study. Although, as noted above, some studies suggest a possible association between PCSK9 genetic variants and an increased risk of type 2 diabetes, there are also studies with contradictory findings. For example, a study has reported pharmacological treatments with PCSK9 inhibitors in patients with primary hypercholesterolemia resulted in increased fasting glucose levels compared to placebo but with no increase in the incidence of diabetes [14]. There is also some discrepant evidence regarding the role of PCSK9 in glucose homeostasis obtained from the studies using PCSK9-deficient mice. In one of these studies conducted by Langhi C et al., they found that PCSK9 deficiency does not affect glucose-stimulated insulin secretion in mouse islets [24], while in the study conducted by Mbikay M et al., PCSK9-null mice showed hyperglycemia and signs of apoptosis in their pancreatic islets [26].

Furthermore, a study conducted by Saavedra et al. found that in patients with autosomal familial hypercholesterolemia, the presence of PCSK9 InsLEU variant (a loss-of-function mutation) was associated with lower LDL cholesterol. However, the prevalence of diabetes and pre-diabetes in these people was twice as high as those without this polymorphism [27]. In contrast, in a study by Bonnefond A et al., the PCSK9 P.R46L variant (the loss-of-function genetic variant) was not associated with impaired glucose homeostasis. These researchers showed that in individuals with the P.R46L variant, the incidence of type 2 diabetes after nine years follow-up or the risk of type 2 diabetes did not increase. They found no relationship between the distribution of genotypic P.R46L variants and fasting glucose, HbA1C, insulin resistance markers (HOMA-IR), or insulin secretion (HOMA-B) [4]. The reasons for these discrepancies are unclear, but the concentration and duration of PCSK9 deficiency may contribute to the increased risk of diabetes and need to be addressed in the future.

## Limitations

There are some limitations in our study to be considered. In the present study, we only focused on the effects of rs615563 variants, the most frequent PCSK9 GOF, while other rare GOF variants of PCSK9 may affect glucose homeostasis. We also did not evaluate the level of PCSK9 and the relationship between circulating PCSK9 and diabetes in the population.

## Supplementary Information

**Additional file 1:** Details of the study population, biochemical analyses, genotyping of rs615563 variants of the methods section.

## Data Availability

The datasets used and/or analyzed during the current study are available from the corresponding author on reasonable request.

## Abbreviations

PCSK9: Proprotein convertase subtilisin/kexin type 9
PCR: Polymerase chain reaction
RFLP: Restriction fragment length polymorphism
NARC1: Neural apoptosis-regulated convertase 1
LDL: Low-density lipoprotein
LDLR: LDL receptor
GOF: Gain-of-function
LOF: Loss-of-function
HDL-C: High-density lipoprotein cholesterol
AST: Aspartate transaminase
ALAT: Alanine aminotransferase
HOMA-IR: Homeostatic Model Assessment for Insulin Resistance
HOMA-B: The HOMA-beta cell function
HbA1c: Hemoglobin A1c
