# Supplementary material for: Association between the rs615563 variant of PCSK9 gene and circulating lipids and Type 2 diabetes
Source: BMC Res Notes. 2021 Aug 11;14:309. doi: 10.1186/s13104-021-05723-4 (PMC8359546; doi:10.1186/s13104-021-05723-4)
Supplement: Supplementary file 1 — Additional file 1: Details of the study population, biochemical analyses, genotyping of rs615563 variants of the methods section. [file 13104_2021_5723_MOESM1_ESM.docx]

**Additional file 1**

**Methods**

**Study population**

The data reported in this study are based on subsamples from a 5-year (2009–2014) cohort study of the adult population of Ahvaz, Iran (590 subjects). The study protocol was approved by the Ethics Committee of Ahvaz Jundishapur University of Medical Sciences (HLRC-9505) and all participants provided written informed consent. Males and females aged 20 years and older were eligible. Individuals with the following conditions were excluded from this study: hepatic or renal diseases, un-treated hypo or hyperthyroidism, major illness such as cancer, use of any lipid-lowering agents. Body mass index (BMI) was calculated by the formula: BMI = kg/m^2^. In this formula, kg is a person's weight in kilograms and m^2^ is their height in meters squared.

**Biochemical Analyses**

Fasting blood samples were drawn from the antecubital vein and then centrifuged at 2500 rpm for 10 min to separate serum. Serum glucose was assayed using an enzymatic colorimetric method based on the hexokinase reaction. Enzymatic colorimetric methods were performed by the BT3000 autoanalyzer to measure AST, ALT, and lipid profile (Pars Azmun kits, Pars Azmun Co., Karaj, Iran). LDL cholesterol and non-HDL cholesterol were calculated using the Friedewald equation and subtracting HDL cholesterol from total cholesterol, respectively. The glycemic status was defined according to the American Diabetes Association 2018 criteria (2018); normal glucose homeostasis was defined as a fasting blood sugar (FBS) level <100 mg/dL without glucose-lowering treatment and all non-diabetic subjects were at normal homeostasis glucose conditions. Diabetes mellitus was defined as FBS ≥126 mg/dL or receiving glucose-lowering agents.

**Genotyping of rs615563 variants**

The rs615563 was genotyped using polymerase chain reaction (PCR) -restriction fragment length polymorphism (RFLP). Genomic DNA was extracted from leukocytes in the EDTA blood samples using a genomic DNA isolation kit (CinnaGen, Iran). PCR was conducted in a final volume of 20 μL, containing 100 ng of genomic DNA, 0.4 μmol/L of each primer (Cinaclone, Iran), 2 X master mixes (Ampliqon, Copenhagen, Denmark) adjusted with nuclease-free water to 20 μL. The sequences of primers were as follow: F: 5’-TGCTCTAATCACGCTCCC-3’ and R :5’- CCATTGGCTAAGAAACCTAAAA-3’. The PCR was conducted according to the following condition: initial activation step at 95 °C for 15 sec, 30 cycles of 94 °C for 30 sec, 60 °C for 30 sec, and 94 °C for 30 sec, and a final extension at 72°C for 3 min. An aliquot of each PCR product was digested with HaeIII (New England Biolabs) at 37 °C for 60 min following the manufacturer's instruction. The digested PCR products were run using agarose gel electrophoresis (3%) and visualized by safe stain (Yektatajhiz Inc., Iran). Then bands were detected using a gel documentation system (Quantum ST4, France).

The amplified product of PCR was a 365bp fragment which was digested to three fragments of 32bp, 333bp, and 365bp. The PCR products with homozygous allele (GG) were digested into two bands of 333bp and 32bp, while the heterozygous (GA) yielded three bands of 365bp, 333bp, and 32 bp, and finally, the homozygous allele (AA) presented one band of 365bp.

**References**

2018. 2. Classification and Diagnosis of Diabetes: Standards of Medical Care in Diabetes-2018. Diabetes Care 41: S13-s27
